# Guidelines for diagnosis and management of the cobalamin-related remethylation disorders cblC, cblD, cblE, cblF, cblG, cblJ and MTHFR deficiency

**DOI:** 10.1007/s10545-016-9991-4

**Published:** 2016-11-30

**Authors:** Martina Huemer, Daria Diodato, Bernd Schwahn, Manuel Schiff, Anabela Bandeira, Jean-Francois Benoist, Alberto Burlina, Roberto Cerone, Maria L. Couce, Angeles Garcia-Cazorla, Giancarlo la Marca, Elisabetta Pasquini, Laura Vilarinho, James D. Weisfeld-Adams, Viktor Kožich, Henk Blom, Matthias R. Baumgartner, Carlo Dionisi-Vici

**Affiliations:** 1 Division of Metabolism and Children’s Research Center, University Childrens’ Hospital Zürich, Zurich, Switzerland; 2 radiz – Rare Disease Initiative Zürich, Clinical Research Priority Program, University of Zürich, Zurich, Switzerland; 3 Department of Paediatrics, Landeskrankenhaus Bregenz, Bregenz, Austria; 4 Division of Metabolism, Bambino Gesù Children’s Research Hospital, Rome, Italy; 5 Willink Biochemical Genetics Unit, Saint Mary’s Hospital, Central Manchester University Hospitals NHS Foundation Trust, Manchester Academic Health Science Centre, Manchester, M13 9WL UK; 6 Reference Center for Inborn Errors of Metabolism, Robert Debré University Hospital, APHP, Paris, France; 7 Inserm U1141, Robert Debré Hospital, Paris, France; 8 Université Paris-Diderot, Sorbonne Paris Cité, site Robert Debré, Paris, France; 9 Metabolic Unit, Centro Hospitalar do Porto, Porto, Portugal; 10 Biochimie, faculté de pharmacie, Université Paris Sud, Paris, France; 11 Division of Inherited Metabolic Diseases, Department of Pediatrics, University Hospital Padova, Padova, Italy; 12 University Dept of Pediatrics, Giannina Gaslini Institute, Genoa, Italy; 13 Congenital Metabolic Diseases Unit, Hospital Clínico Universitario de Santiago de Compostela, IDIS, CIBER, Compostela, Spain; 14 Department of Neurology, Neurometabolism Unit, and CIBERER (ISCIII), Hospital Sant Joan de Deu, Barcelona, Spain; 15 Department of Experimental and Clinical Biomedical Sciences, University of Florence, Firence, Italy; 16 Metabolic and Newborn Screening Clinical Unit, Department of Neurosciences, A. Meyer Children’s University Hospital, Florence, Italy; 17 Newborn Screening, Metabolism & Genetics Unit, National Institute of Health, Porto, Portugal; 18 Section of Clinical Genetics and Metabolism, Department of Pediatrics, University of Colorado School of Medicine, Aurora, CO USA; 19 Inherited Metabolic Diseases Clinic, Childrens Hospital Colorado, Aurora, CO USA; 20 Institute of Inherited Metabolic Disorders, Charles University-First Faculty of Medicine and General University Hospital, Prague, Czech Republic; 21 Laboratory of Clinical Biochemistry and Metabolism, Center for Pediatrics and Adolescent Medicine University Hospital, Freiburg, Freiburg, Germany

## Abstract

**Background:**

Remethylation defects are rare inherited disorders in which impaired remethylation of homocysteine to methionine leads to accumulation of homocysteine and perturbation of numerous methylation reactions.

**Objective:**

To summarise clinical and biochemical characteristics of these severe disorders and to provide guidelines on diagnosis and management.

**Data sources:**

Review, evaluation and discussion of the medical literature (Medline, Cochrane databases) by a panel of experts on these rare diseases following the GRADE approach.

**Key recommendations:**

We strongly recommend measuring plasma total homocysteine in any patient presenting with the combination of neurological and/or visual and/or haematological symptoms, subacute spinal cord degeneration, atypical haemolytic uraemic syndrome or unexplained vascular thrombosis. We strongly recommend to initiate treatment with parenteral hydroxocobalamin without delay in any suspected remethylation disorder; it significantly improves survival and incidence of severe complications. We strongly recommend betaine treatment in individuals with MTHFR deficiency; it improves the outcome and prevents disease when given early.

**Electronic supplementary material:**

The online version of this article (doi:10.1007/s10545-016-9991-4) contains supplementary material, which is available to authorised users.

## Introduction

This guideline development process was initiated within the frame of the “European network and registry for homocystinurias and methylation defects” (E-HOD) project, which was started in February 2013. The main aims of the project are the formation of a sustainable international collaboration of experts and clinically active centres, development of guidelines and establishment of a disease registry for homocystinurias and methylation defects. Given the often significant delays in diagnosis and the absence of standardised treatment protocols, the evaluation of the published knowledge and delineation of guidelines for diagnosis and treatment of these rare diseases are urgently needed. Most of the existing studies and reports are non-systematic, observational studies, case series or case reports, which are generally considered to be low quality evidence. However, following collation of the available evidence some very consistent patterns evolved. Confirmation and validation of our observations by insights gained from other fields of research (e.g. vitamin B12 deficiency in the elderly) additionally informed our interpretation of the evidence.

Homocysteine (Hcy) is an amino acid formed from methionine (Met). Under normal circumstances Hcy is converted into cysteine (transulfuration pathway) or remethylated (remethylation pathway) forming Met.

**Fig. 1:**
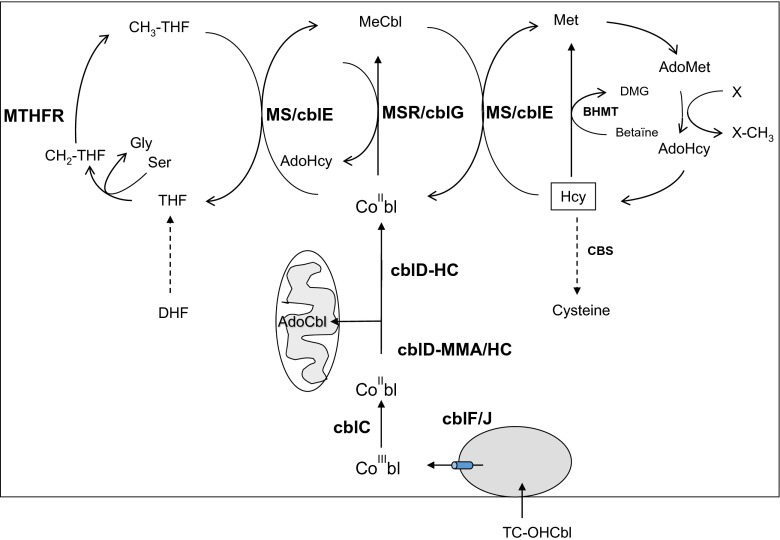
Remethylation disorders: metabolic pathways. *MTHFR* methylenetetrahydrofolate reductase, *THF* tetrahydrofolate, *Gly* glycine, *Ser* serine, *DHF* dihydrohydrofolate, *Met* methionine, *Hcy* homocysteine, *AdoMet* adenosylmethionine, *AdoHcy* adenosylhomocysteine, *MS* methionine synthase, *MSR* methionine synthase reductase, *BHMT* betaine homocysteine methyltransferase, *CBS* cystathionine-beta-synthase, *DMG* dimethylglycine, *MeCbl* methylcobalamin, *AdoCbl* adenosyl-Cbl

All genetic remethylation defects share deficient activity of methionine synthase due to various reasons: decreased function of the methionine synthase enzyme protein itself or of the associated enzyme methionine synthase reductase; deficient production of the cofactor, methyl-cobalamin; or disturbed supply of the substrate methyl-tetrahydrofolate (MTHF). Some genetic disorders of intracellular cobalamin (cbl) transport and processing (cblC, cblD-MMA/Hcy, cblF and cblJ) cause deficient synthesis not only of methylcobalamin but also of adenosylcobalamin, the cofactor for methylmalonyl-CoA mutase. Those combined remethylation disorders are associated with increased Hcy and methylmalonic acid (MMA). 5,10-methylenetetrahydrofolate reductase (MTHFR) deficiency leads to impaired provision of 5-MTHF resulting in decreased function of methionine synthase (Fowler 2005) (Fig. 1)

.

Our understanding of the pathophysiology of the remethylation disorders and MTHFR deficiency is incomplete. At present, there are five main hypotheses: (A) direct toxicity of metabolites, (B) missing products, (C) impaired methylation capacity, (D) oxidative stress, (E) impaired non-enzymatic protein functions.

(A) Hyperhomocysteinaemias are predominantly associated with neurocognitive and vascular pathology. Homocysteine and its metabolic product, homocysteic acid, have been implicated in provocation of seizures in rats (Kubová et al 1995; Mares et al 1997). Besides its impact on renal function, accumulation of MMA along with dicarboxylic acids like 2-methylcitrate and propionyl-CoA have been found to induce synergistic mitochondrial dysfunction (Morath et al 2008; Zsengellér et al 2014).
(B) Impaired methionine synthase activity with accumulation of intracellular 5-MTHF (folate trap) and subsequent disruption of nucleotide synthesis compromise rapidly proliferating tissues such as bone marrow or epithelia. In MTHFR deficiency, this trapping of folates is not found but 5-methylTHF synthesis is impaired, resulting in low levels of methyl-tetrahydrofolate (CH3-THF) in the central nervous system of unknown significance (Schiff and Blom 2012).
(C) Remethylation of homocysteine (Hcy) is an important mechanism that maintains the methylation capacity of the organism and removes excess Hcy. Methylation reactions such as DNA methylation, creatine and myelin synthesis utilise S-adenosylmethionine (SAM), a metabolite formed from Met as the methyl group donor. Accordingly, any disorder or disruption of the Met-Hcy-SAM pathway affects methylation capacity by impairing multiple metabolic systems and processes (King et al 2012; Surtees et al 1991).
(D) Enhanced oxidative stress due to a disturbance of glutathione metabolism has been shown in patients with the cblC defect and may contribute to the underlying pathophysiology (Pastore et al 2014).
(E) Recent data suggest that not only the accumulation of metabolites but also the perturbed non-enzymatic functions, e.g. of the MMACHC protein may add to the pathophysiology behind the disease (Moreno-Garcia et al 2014; Brooks et al 2016).

## Methods

Panelists involved in the guideline process were invited by the EHOD project leaders according to their expertise regarding the diseases in terms of biochemistry; genetics; clinical, psychosocial and laboratory diagnosis and patient management and/or research activities.

The PubMed and Cochrane databases were searched using the following terms: remethylation defects OR severe mthfr OR severe methylenetetrahydrofolate reductase OR cblc OR cbld OR cblf OR cblj OR cble OR cblg OR methylmalonic aciduria homocystinuria OR methylmalonic aciduria homocystinuria OR methylmalonic acidemia homocysteinemia OR methylmalonic aciduria homocysteinemia OR “Cobalamin C OR Cobalamin D OR Cobalamin E OR Cobalamin F OR Cobalamin G OR Cobalamin J” (last update February 2016: 834 publications).

The resulting list of publications was disposed of all articles predominantly or only addressing other diseases (e.g. classical homocystinuria) or MTHFR polymorphisms (e.g. C677T) or without information relevant to the outcomes of interest (e.g. mutation reports only) by screening of abstracts. In total, 174 publications were included in the qualitative data analysis. The clinical characterisation of the diseases (Table 1) was based on 117 publications.

**Table 1:** Signs and symptoms reported in 396 individuals. ++++ Very frequently (>50 % of cases); +++ frequently (25–50 % of cases); ++ infrequently (10–25 % of cases); + occasionally seen (<10 % of cases). (+) Single case reports, probably disease-related conditions

	cblC	cblD-MMA/HC	cblF	cblD-HC	cblE	cblG	MTHFR deficiency
Number of reported cases	169	16	13	5	20	25	148
Eating disorders/failure to gain weight							
Small for gestational age	+		++				(+)
Feeding difficulties, failure to thrive	+++	++++	+++		+++	++++	++
Nervous system							
Decreased consciousness +/− apnoea	++	(+)			+++	+++	+++
Seizures	+++	++++	++	(++)	+++	+++	+++
Ataxia	+			(++)	(+)	++	++
Movement disorder and/or abnormal muscle tone	+++	+++	+++	++++	+++	++++	+++
Peripheral neuropathy/subacute degeneration of spinal cord	++	(+)		(++)	++	++	++
Hydrocephalus	++				++	++	++
Visual impairment (retinopathy, optic atrophy)	+++	++		+++	++	++	+
Developmental disorder/ cognitive impairment	+++	+++	++++	++++	++++	++++	++++
Behavioural/mental disorders	++	++		++++		(+)	++
Microcephaly	++			++++	++	+	++
Blood and bone marrow							
Megaloblastic anaemia	++	++	+++	+++	++++	++++	+
Pancytopenia/neutropenia	++		+++		++	+	
Recurrent severe infections	(+)		++				
Kidneys							
Haemolytic uraemic syndrome	++	++			++	(+)	
Glomerulopathy	+				(+)		
Tubulointerstitial nephropathy	+						
Cardiopulmonary							
Cardiac malformation	+		+++				
Cardiomyopathy	++						
Interstitial pneumonia	+						
Pulmonary hypertension	+						
Vascular							
Stroke	(+)				(+)		+
Venous thrombosis/ embolism	+					(+)	+
Malformations							
Facial dysmorphism	+					(+)	
Skeletal deformity	(+)						+
Gastrointestinal							
Cheilitis/gastritis			+++				
Liver steatosis	+		++				
Skin							
Dermatitis/rash/hyperpigmentation	+		++				+
Other							
Hydrops fetalis	+						
Metabolic acidosis and/or hyperammonaemia	+						
Temperature instability/hypothermia	+						(+)

Legend: The frequencies depicted in Table 1 are derived from collated case reports and may not be fully representative. However, they do not significantly deviate from published case series (Ogier de Baulny et al 1998; Carrillo-Carrasco et al 2012; Fischer et al 2014; Huemer et al 2016). The true prevalence of organ manifestations also depends on the extent of technical investigations, especially in relation to milder forms of microangiopathy and retinopathy that may escape clinical detection. Due to the very small number of reported cblJ cases, these have not been included

References: Fischer et al 2014; Al Essa et al 1999; Al Tawari et al 2002; Alfadhel et al 2011; Andersson et al 1999; Andersson and Shapira 1998; Arai and Osaka 2011; Arn et al 1998; Atkinson et al 2014; Augoustides-Savvopoulou et al 1999; Backe et al 2013; Baumgartner et al 1979a; Baumgartner et al 1979b; Beauchamp et al 2009; Bellini et al 1992; Ben-Shachar et al 2012; Biancheri et al 2002; Biancheri et al 2001; Biotti et al 2014; Birnbaum et al 2008; Bishop et al 2008; Brandstetter et al 1990; Broomfield et al 2014; Brunel-Guitton et al 2010; Brunelli et al 2002; Cappuccio et al 2014; Carmel et al 1980; Carmel et al 1988; Carrillo-Carrasco et al 2009; Carrillo-Carrasco et al 2012a, b; Cerone et al 2000; Chang et al 2011; Clayton et al 1986; Coelho et al 2008; Cogan et al 1980; D’Aco et al 2014; D’Alessandro et al 2010; De Bie et al 2009; Dionisi-Vici et al 2013; Ellaway et al 1998; Engelbrecht et al 1997; Enns et al 1999; Fuchs et al 2012; Geraghty et al 1992; Gerth et al 2008; Goodman et al 1970; Goyette et al 1996; Goyette et al 1995; Goyette et al 1994; Grant et al 2009; Grünert et al 2011; Guigonis et al 2005; Gulati et al 1997; Haan et al 1985; Harding et al 1997; Harpey et al 1981; Haworth et al 1993; Holme and Ronge 1989; Howard et al 1997; Huemer et al 2005, 2014, 2015a, b; Hyland et al 1988; Kanwar et al 1976; Kind et al 2002; Lesesve and Latger-Cannard 2013; Levy et al 1970; Longo et al 2005; Lossos et al 2014; Mah et al 2013; Martinelli et al 2011; Matos et al 2013; Menni et al 2012; Michot et al 2008; Mitchell et al 1986; Morel et al 2005; Mudd and Freeman 1974; Nishimura et al 1985; Ogier de Baulny et al 1998; Pasquier et al 1994; Patton et al 2000; Paul et al 2013; Powers et al 2001; Prasad et al 2011; Profitlich et al 2009a, b; Regland et al 1994; Ribes et al 1990; Ricci et al 2005; Ronge and Kiellman 1996; Rosenblatt et al 1985; Rosenblatt et al 1986; Rossi et al 2001; Rutsch et al 2009; Schiff et al 2011; Schimel and Mets 2006; Selzer et al 2003; Shih et al 1989; Shinnar and Singer 1984; Sibani et al 2000; Smith and Bodamer 2002; Steen et al 1997; Stucki et al 2012; Suormala et al 2004; Tanpaiboon et al 2013; Tomaske et al 2001; Tonetti et al 2003; Traboulsi et al 1992; Ucar et al 2010; Urbón Artero et al 2002; Urreizti et al 2010; Visy et al 1991; Waggoner et al 1998; Wang et al 2014; Watkins and Rosenblatt 1989; Wu et al 2005

The following main questions and outcomes of interest in males and females with any type of onset (early or late) of one of the diseases of interest were addressed (outcomes in parentheses).

- Which clinical signs are characteristic and allow timely diagnosis (Outcome: timely clinical diagnosis; this outcome is considered important)
- Which biochemical parameters allow timely and valid diagnosis? (Outcome: valid, timely laboratory diagnosis; this outcome is considered important)
- How can we prevent death and avoid/treat severe organ damage (Outcome: survival, severe organ complications; this outcome is considered critical)
- How can we prevent or treat eye disease and neurocognitive impairment? (Outcome: visual and neurocognitive function; this outcome is considered critical)

In a first step, all reports made available to the panelists were graded according to the Scottish Intercollegiate Guidelines Network (SIGN) criteria by the authors who had assigned themselves according to their expertise to five major working groups focusing on the topics “clinical signs and symptoms”; “differential diagnosis”; “laboratory/biochemical parameters”; “disease course and outcome”; “treatment”. Due to the rarity of the diseases, studies and reports of all types of designs (from case reports and case series to metaanalyses) were included; randomised controlled trials were not available.

The working groups prepared drafts on their topics, which were carefully evaluated in moderated discussion groups. Elaboration and grading of recommendations was accomplished by moderated, consensus-oriented discussions according to the “Grading of Recommendations Assessment, Development and Evaluation (GRADE) working group” approach (http://www.gradeworkinggroup.org/; Guyatt et al 2011a, b, c, 2012); a patient representative was present at these meetings. Finally, for each outcome the quality of the evidence was rated in one of three categories (low, moderate, high) as defined by the GRADE working group. The strength of a recommendation (strong recommendation, recommendation and suggestion; for more information see Supplementary material) was summarised from written assessments from the panelist using the following set of questions:

- Are you confident that the benefits outweigh the harms/burden or vice versa?
- Is there high, moderate or low quality evidence? Please consider:
  - Risk of bias
  - Study design
  - Directness and consistency of results
  - Magnitude of effect
  - Dose–response gradient
  - Publication bias
- Are you confident that the recommendation meets typical values and preferences of the target population (e.g. patients, parents)?
- Are the resources worth the expected net benefit following the recommendation?
- Overall strength of recommendation: weak or strong?

Data derived from case reports, case series or any other type of observational studies were by principle rated as low quality evidence. However, evidence for some outcomes was considered moderate on the basis of high consistency of results. High quality of evidence was only available for betaine treatment in MTHFR deficiency. This evidence was based on a metaanalysis, which revealed direct and consistent results as well as a relationship between time of onset of treatment and outcome.

## Which clinical signs are characteristic for remethylation defects and allow for timely diagnosis?

### General clinical patterns of remethylation disorders

The published clinical presentations of patients suffering from remethylation disorders (summarised in Table 1) reveal no specific, distinct signs and symptoms but rather highlight that these conditions affect multiple systems and thus often present in a rather multifaceted manner. Nevertheless, a few patterns of clinical presentations can be identified.

Most prominently, the central and peripheral nervous system and the bone marrow are affected. Developmental and neurocognitive impairment, feeding problems, neurological symptoms including seizures, movement disorders, abnormal muscle tone, visual impairment, neuropathy and haematological abnormalities are present in the majority of patients. In many patients, renal manifestations, e.g. atypical haemolytic uraemic syndrome (HUS) or glomerulopathy, mostly related to microangiopathy, have been identified.

Clinical presentations may vary considerably. Severe acute encephalopathy, macrocytic anaemia, atypical HUS, cardiopulmonary signs, subacute combined degeneration of the cord or psychiatric symptoms, each isolated or in combination with other symptoms may be present. Therefore, in particular, a combination of neurological and haematological symptoms, often in the presence of failure to thrive or feeding difficulties should raise suspicion of a remethylation disorder (see references to Table 1).

### Age specific patterns of remethylation disorders

Findings from published case reports indicate that the patterns of clinical manifestation of remethylation disorders vary with age (**Table** 2).

**Table 2:** Age-related clinical presentations of remethylation disorders

Neonates (0–28 days)
Encephalopathy
Lethargy, apnoea
Feeding difficulties
Muscular hypotonia
Seizures
Nystagmus
Anaemia/thrombocytopenia or pancytopenia, megaloblastosis
Haemolytic uremic syndrome
Cardiomyopathy
Hydrocephalus
Pulmonary hypertension
Infants (1–12 months)
Growth failure/poor weight gain
Acute progressive encephalopathy/apnoea
Chronic encephalopathy
Muscular hypotonia
Developmental disability/regression
Seizures
Recurrent acute behavioural changes/lethargy
Visual inattention/Nystagmus
Anaemia/thrombocytopenia or pancytopenia, megaloblastosis
Haemolytic uremic syndrome
Pulmonary hypertension
Children (1–12 years)
Chronic Encephalopathy
Muscular hypotonia or spasticity
Developmental disability/regression or dementia
Seizures
Neuropsychiatric disturbance/personality changes
Intermittent acute behavioural changes/lethargy
Acute progressive encephalopathy/apnoea
Subacute degeneration of the cord
Paraesthesia
Incontinence
Ataxia/spasticity
Progressive limb weakness (legs>arms)
Haemolytic uremic syndrome
Thromboembolic events
Recurrent venous thrombosis
Pulmonary thromboembolism
Cerebrovascular events
Pulmonary hypertension
Adolescents and adults (>12 years)
Chronic encephalopathy
Developmental disability/regression or dementia
Neuropsychiatric disturbance
Personality changes
Intermittent acute behavioural changes/lethargy
Acute progressive encephalopathy
Subacute degeneration of the spinal cord
Paraesthesia
Incontinence
Ataxia/spasticity
Progressive limb weakness (legs>arms)
Thromboembolic events
Recurrent venous thrombosis
Pulmonary thromboembolism
Cerebrovascular events
Pulmonary hypertension

### Clinical patterns specific for combined remethylation disorders (cblC, cblD-MMA/HC, cblF, cblJ)

Severe intrauterine growth retardation is rare, whereas postnatal growth failure and feeding difficulties are very frequent. Three quarters of children with combined disorders manifest during the neonatal period or in early infancy. Neonates typically show lethargy, seizures and muscular hypotonia, often in combination with megaloblastic anaemia or neutropenia/pancytopenia. Up to 50 % of infants exhibit signs of visual impairment such as nystagmus on the background of retinopathy or optic atrophy (Bonafede et al 2015; Weisfeld-Adams et al 2015; Brooks et al 2016). Microangiopathic renal or pulmonary disease mostly manifests early but may also be present in older children or adults (Grangé et al 2015; Kömhoff et al 2013; Sharma et al 2007).

Older infants and young children often show signs of acute encephalopathy; visual and cognitive impairment are prevalent. Older children, adolescents and adults may present with acute or chronic behavioural or psychiatric abnormalities, cognitive impairment, signs of peripheral neuropathy and ataxia that reflect subacute degeneration of the spinal cord or rarely venous thromboembolism (Rahmandar et al 2014; Huemer et al 2014; Grangé et al 2015). Other than optic pallor, ocular manifestations are rare in late-onset cblC (Gizicki et al 2014; Weisfeld-Adams et al 2015).

### Clinical patterns specific for isolated remethylation disorders (cblD-HC, cblE, cblG)

The less prevalent isolated disorders of methionine synthase generally present similar to combined disorders. The typical clinical patterns of the cblE and the cblG defect are indistinguishable. Affected individuals usually manifest in infancy, mostly with haematological abnormalities, muscular hypotonia and neurocognitive impairment. One third of all patients present with impaired consciousness, seizures, and one quarter with signs of visual impairment. Older children and adults can manifest with signs reflecting spinal cord involvement (subacute degeneration of the spinal cord) or psychiatric symptoms (Huemer et al 2015a). Single cases with isolated macrocytic anaemia without neurocognitive impairment have been reported (Vilaseca et al 2003; Ruiz-Mercado et al 2015).

### Clinical patterns specific for MTHFR deficiency

MTHFR deficiency mainly presents in early childhood but can potentially present at any age with a high prevalence of cognitive impairment. Neonates and young infants often manifest with feeding difficulties, decreased consciousness, hydrocephalus and muscular hypotonia. Apnoea is a frequent complication (Broomfield et al 2014; Diekman et al 2014; Huemer et al 2016; Schiff et al 2011; Strauss et al 2007).

Older infants and children frequently manifest with seizures and cognitive impairment, often displaying acquired microcephaly. With increasing age, peripheral neuropathy, gait abnormalities and spasticity may become evident and some older patients manifest with additional or even isolated behavioural or psychiatric disorders (Birnbaum et al 2008; Michot et al 2008).

Individuals with MTHFR deficiency do not usually present with haematological abnormalities. There are just a few reports of older children and adults with megaloblastic anaemia in association with long-standing neurological manifestations (Broomfield et al 2014; Schiff et al 2011).

**Outcome: timely clinical diagnosis**

1. We strongly recommend consideration of an acquired or genetic disorder of remethylation in the case of neurological and/or visual and/or haematological symptoms. (Quality of the evidence: moderate)
2. We strongly recommend considering an acquired or genetic disorder of remethylation in the case of unexplained thrombosis and/or spinal cord degeneration and/or atypical HUS. (Quality of the evidence: moderate)

### Clinical differential diagnosis

All clinical presentations of genetic disorders of remethylation can be mimicked by common conditions, which reduce the availability of folate or cobalamin such as nutritional deficiency, acquired malabsorption or drug-associated effects (Table 3). Maternal vitamin B12 deficiency due to a strict vegan diet or to undiagnosed pernicious anaemia needs to be excluded in neonates and young infants presenting with signs of a remethylation disorder or identified by newborn screening (NBS) and subnormal vitamin B12 concentrations (Scolamiero et al 2014).

**Table 3:** Conditions that mimic intracellular disorders of remethylation

Affecting cobalamin availability
Nutritional inadequacy (maternal vitamin B12 deficiency/vegan diet)
Intestinal malabsorption (e.g. genetic disorders such as Imerslund-Graesbeck syndrome gastric intrinsic factor deficiency; autoimmune or parasitic disease; short gut syndrome)
Disturbed binding and cellular uptake (e.g. TC deficiency)
Disturbance of intracellular metabolism (nitrous oxide)
Affecting folate availability
Nutritional inadequacy (maternal deficiency/dietary inadequacy)
Intestinal malabsorption (acquired/genetic)
Disturbed binding and cellular uptake of folate (autoimmune/genetic)
Disturbance of intracellular metabolism (antifolate drugs, MTHFD1 deficiency)
Other diseases with a combination of haematological and neurological symptoms
Severe iron deficiency
Infectious diseases (e.g. CMV/EBV/HHV6/HPVB19/HIV1)
Leukaemia/myeloproliferative disorders
Myelodysplastic syndromes
Disorders affecting the mitochondrial respiratory chain (e.g. Pearson syndrome)
UMP synthase deficiency (orotic aciduria)
Thiamine transporter (SLC19A2) defect
Branched chain organic acidaemia such as methylmalonic or propionic acidaemia
Lysinuric protein intolerance

A number of other rare genetic diseases can mimic defects in intracellular cobalamin metabolism and should be considered in children with clinical signs and biochemical hallmarks of a remethylation disorder but with normal serum concentrations of vitamin B12 and folates: transcobalamin (TC) deficiency is biochemically and clinically indistinguishable from combined cobalamin disorders (Trakadis et al 2014). The recently discovered MTHFD1 deficiency (Watkins and Rosenblatt 2012) affects folate metabolism and can cause mild hyperhomocysteinaemia. The five patients described so far displayed severe megaloblastic anaemia and pancytopenia, immunological problems and renal microangiopathy (Burda et al 2015).

The HCFC1 X-linked defect (cbl X, Yu et al 2013) is sometimes associated with disturbed function of the CblC protein but its diagnosis can be challenging because not all affected boys have elevated homocysteine concentrations. Clinical hallmarks are neonatal or early infantile onset of severe developmental disorder, prominent epileptic seizures, cognitive impairment and a dystonic-choreatic movement disorder; moderately elevated MMA was observed in all 14 patients reported by Yu et al 2013 (Table 4).

**Table 4:** Differential diagnosis of inborn errors of metabolism presenting with hyperhomocysteinaemia

	Macrocytosis or macrocytic anaemia	MMA	Met	Total vitamin B12	Folate
cblC cblD-MMA/HC	+ or −	↗	↘↘ to nl	nl	nl
cblF/cblJ	+	↗	↘↘ to nl	nl	nl
cblE/G	+	nl	↘↘ to nl	nl	nl
cblD-HC	+ or −	nl	↘↘ to nl	nl	nl
MTHFR	–	nl	↘↘ to nl	nl	nl or ↘
Vitamin B12 deficiency or malabsorption	+	↗	↘ to nl	↘↘	nl
Folate deficiency or malabsorption	+	nl	↘ to nl	nl	↘↘
HCFC1 (cblX)	+ or −	↗ or nl	↘↘ to nl	nl	nl
CBS deficiency	–	nl	nl-↗	nl	nl
TC deficiency	+	↗	↘↘ to nl	nl (↘)	nl
MTHFD1 deficiency*	+	nl	↘↘ to nl	nl	nl

*Hyperhomocysteinaemia present in some patients, normal (nl) tHcy levels in others

## Which parameters allow valid and timely laboratory diagnosis?

**Fig. 2:**
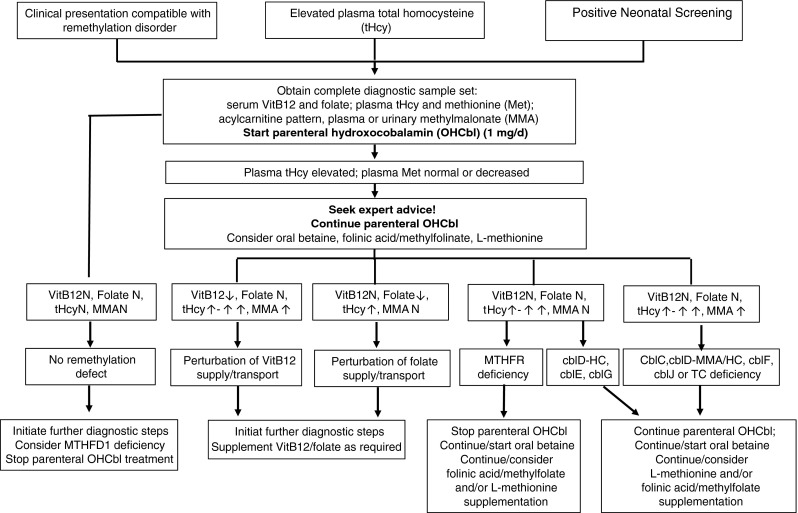
Diagnostic pathway and management of the patient with a suspected remethylation disorder. All biochemical results refer to pre-treatment samples. **N = normal**

Patients with a suspected remethylation disorder require immediate and urgent biochemical investigation (Fig. 2). The diagnosis of remethylation disorders can be confirmed by investigations at the levels of metabolites, enzymatic studies and/or molecular genetic analysis.

### Biochemical metabolites

Plasma total homocysteine (tHcy) is the first biochemical parameter to assess when a remethylation disorder is suspected. THcy levels usually are >50 but mostly >100 μmol/L in untreated patients with remethylation disorders (Carrillo-Carrasco et al 2009; Van Hove et al 2002; D’Aco et al 2014; Miousse et al 2009).

Routinely, tHcy is measured by chromatographic methods or immunoassays. Chromatographic methods are more specific than immunoassays and are usually coupled with tandem mass spectrometry (MS/MS) detection allowing simultaneous determination of other compounds of interest for diagnosis and follow up of these diseases, e.g. Met, cysteine (Refsum et al 2004) or possibly cystathionine (Stabler et al 1993; Stabler et al 2013).

There is no influence of the type of collection tube used (EDTA, heparinised, citrate, serum, gel separator tubes). However, blood samples should be centrifuged within 1 h or kept cold until centrifugation (<6 h). After removal of blood cells, tHcy is stable for several weeks at 4 °C and for several years after freezing (Refsum et al 2004).

Free homocystine in plasma or urine measured by conventional ion-exchange chromatography is not the method of choice since it may remain undetectable even in the presence of significantly elevated tHcy in plasma (Fowler and Jakobs 1998; Moat et al 1999).

To further discriminate remethylation disorders from other hyperhomocysteinaemias, determination of urinary (or plasma) MMA (Fowler and Jakobs 1998), plasma Met, blood acylcarnitine profile, serum vitamin B12 and folate are required and should promptly be determined (see Figs. 1 and 2). In case shipment of urine or blood samples is difficult, an experienced laboratory should give advice on the investigation of the parameters from DBS. Treatment should not be delayed by waiting for confirmation of the exact defect.

**Outcome: valid, timely laboratory diagnosis**

Recommendation 3
  Elevated plasma tHcy is the hallmark of remethylation disorders. We strongly recommend that investigations in patients with a suspected remethylation disorder should start with the measurement of total homocysteine in blood. We recommend the blood sample for tHcy to be centrifuged within an hour and kept at +4° or frozen until analysis. Immunoassays or chromatographic methods are suitable for tHcy measurement. (Quality of the evidence: moderate)
Recommendation 4
  We strongly recommend against measuring free homocysteine instead of total homocysteine. (Quality of the evidence: moderate)
Recommendation 5
  We strongly recommend that in the case of high total homocysteine, plasma and urine samples for determination of MMA, methionine, folate and vitamin B12 are to be obtained before treatment is started. (Quality of the evidence: moderate)

### Differential diagnosis of biochemical parameters

Hyperhomocysteinaemia is the hallmark of the remethylation and transulfuration disorders. Mild to moderate hyperhomocysteinaemia is common in vitamin B12 or folate deficiencies but also in very severe vitamin B6 deficiency and in patients with renal failure or hypothyroidism. While megaloblastic anaemia with increased MCV and hypersegmented neutrophils on blood film are found in both vitamin B12 and folate deficiencies, elevated MMA is only observed in functional vitamin B12 deficiency (Whitehead 2006).

In the case of normal serum vitamin B12 and folate levels, TC deficiency, primary remethylation and transulfuration disorders should be considered. Holotranscobalamin measurement, which unfortunately is performed only in a few laboratories shows low levels in TC deficiency; this disorder can be confirmed by molecular genetics investigation (Trakadis et al 2014).

The plasma Met concentration differentiates between remethylation disorders and deficiency of CBS, a key enzyme of the transulfuration pathway. Methionine is low or normal in remethylation disorders while it is usually elevated or at least borderline normal in CBS deficiency (Mudd 2011).

Megaloblastic anaemia with increased MCV is often observed in the neonatal forms of intracellular cobalamin defects (cblF, cblJ, cblC, cblD-MMA/HC, cblD-HC, cblE and cblG) but less frequently in the late onset forms of these disorders and not normally in MTHFR deficiency (Rosenblatt et al 1997; Carrillo-Carrasco et al. 2013).

The recently described HCFC1 defect (cbl X) has a different pathophysiological background and is beyond the scope of these guidelines. All 14 patients presented with moderately increased MMA and five of these patients had hyperhomocysteinaemia (Yu et al 2013). In two of the five published patients with methylenetetrahydrofolate dehydrogenase 1 deficiency, tHcy concentrations were moderately elevated (Burda et al 2015) (Table 4).

### Enzymatic studies

Direct enzyme assays are only available for MTHFR, methionine synthase and methionine synthase reductase (Suormala et al 2002; Gulati et al 1997; Olteanu and Banerjee 2001). These direct enzyme assays can be problematic. MTHFR should be assayed in cultured skin fibroblasts preferably in the physiological direction of the reaction (Suormala et al 2002). Apo-methionine synthase and holo-methionine synthase (methionine synthase plus methionine synthase reductase) activities can be measured by the same reaction but with different conditions of reduction (Yamada et al 2006).

Indirect assays measuring the integrity of several enzymes in the same pathway are useful for remethylation disorders investigations. Incorporation of 1-[^14^C]-propionate into amino acids and then cell proteins via the propionate pathway reflects the level of intracellular synthesis of adenosylcobalamin. Incorporation of [^14^C]-methyltetrahydrofolate into methionine explores the methionine synthase and methionine synthase reductase activities and the capacity of the cells to produce methylcobalamin. Incorporation of [^14^C]-formate into methionine or serine reflects the activities of the methylene-tetrahydrofolate dehydrogenase, methylene-tetrahydrofolate reductase and the methionine synthase/methionine synthase reductase activities (Table 5). These tests also allow the assessment of possible in vitro vitamin-responsiveness of the relevant disorders (Suormala et al 2002; Gulati et al 1997; Olteanu and Banerjee 2001). Enzyme assays are needed to characterise functional consequences of new variants identified by molecular genetic investigations including next generation sequencing.

**Table 5:** Representation of the cobalamin defects associated with hyperhomocysteinaemia, the respective enzymatic/incorporations tests available and the genes involved in these diseases

	cblC	cblD-MMA/HC	cblF	cblJ	cblD-HC	cblE	cblG	MTHFR
Direct enzyme assay (tissues)	no	no	no	no	no	yes	yes	yes
						fib/leuc/amn	fib/leuc/amn	fib/leuc/amn
Indirect enzyme assays								
Propionate incorporation	↘	↘	↘	↘	nl	nl	nl	nl
MTHF incorporation	↘	↘	↘	↘	↘	↘	↘	nl
Formate incorporation into serine	↘	↘	↘	↘	↘	↘	↘	nl or ↗
Formate incorporation into methionine	↘	↘	↘	↘	↘	↘	↘	↘
AdoCbl biosynthesis	↘	↘	↘	↘	nl	nl	nl	nl
MeCbl biosynthesis	↘	↘	↘	↘	↘	↘	↘	↘
Gene	***MMACHC*** ^*1*^	*MMADHC* ^*2*^	*LMBRD1* ^*3*^	*ABCD4* ^*4*^	*MMADHC* ^*2*^	*MTRR* ^*5*^	*MTR* ^*6*^	*MTHFR* ^*7*^
Chromosome location	1p34.1	2q23.2	6q13	14q24.3	2q23.2	5p15.31	1q43	1p36.22
Mode of inheritance	AR	AR	AR	AR	AR	AR	AR	AR
OMIM	609831	611935	612625	603214	611935	602568	156570	607093

*fib* fibroblasts, *leuc* leukocytes, *amn* amniocytes, *AR* autosomal recessive, *nl* normal

^1^Lerner-Ellis et al 2006; ^2^Coelho et al 2008; ^3^Rutsch et al 2009; ^4^Coelho et al 2012; ^5^Leclerc et al 1998; ^6^Li et al 1996; ^7^Goyette et al 1994, 1995

### Molecular genetic analysis

The cblC gene (*MMACHC* OMIM 609831) is located in chromosome region 1p34.1 and has five exons (Lerner-Ellis et al 2006). The three most common mutations c.271dupA, c.394C>T and c.331C>T have been identified in the *MMACHC* gene. The c.271dupA that represents about 30 % of the identified alleles and c.331C>T mutations are associated with early onset disease. The c.394C>T mutation is associated mainly with late onset disease. The c.609G>A mutation accounts for 85 % of the identified alleles in Chinese patients, and even though it leads to a premature termination codon it remains predominantly associated with late onset disease in this population (Wang et al 2010) but has been observed in some early-onset cases in other populations (Weisfeld-Adams et al 2013). Other mutations have been shown to cluster according to ethnicity (Morel et al 2006; Nogueira et al 2008).

The precise function of MMACHC is not completely understood. Recombinant MMACHC binds cobalamin, can function in vitro as a cyanocobalamin (CNCbl) decyanase (Kim et al 2008) and can dealkylate other cobalamin forms (Hannibal et al 2009). MMACHC may act as an intracellular cobalamin trafficking chaperone that carries out targeted delivery of cobalamin to and from other cobalamin-related proteins (Lerner-Ellis et al 2009).

In contrast to the cblC defect which presents exclusively as combined hyperhomocysteinaemia with MMA-uria, cblD patients can have three distinct biochemical phenotypes: isolated hyperhomocysteinemia (cblD-HC), isolated MMA-uria (cblD-MMA, beyond the scope of these guidelines), or combined hyperhomocysteinemia and MMA-uria (cblD-MMA/HC). *MMADHC* (OMIM 611935) is the gene responsible for cblD defect (Suormala et al 2004; Coelho et al 2008). Genotype–phenotype correlation analysis of the three cblD variants suggests that the N- and C-terminal regions of *MMADHC* have specific effects on functions in the mitochondrion and cytoplasm, respectively (Stucki et al 2012; Miousse et al 2009). cblD-HC retains mitochondrial function with a full-length protein which harbours only C-terminal missense mutations in conserved residues; cblD-MMA retains cytoplasmic function due to translation of an error-free C-terminus facilitated by downstream re-initiation; and in cblD-MMA/HC complete loss of functionality occurs with deleterious mutations that are localised downstream of Met116 (Jusufi et al 2014).

To date, there have been no obvious genotype-phenotype correlations in cblF (*LMBRD1*, OMIM 612625) (Armour et al 2013), cblJ (*ABCD4*, OMIM 603214) (Coelho et al 2012), cblE (*MTRR*, OMIM 602568) and cblG (*MTR*, OMIM 156570) (Watkins and Rosenblatt 1989; Huemer et al 2015a).

If clinical and biochemical parameters are characteristic for a combined remethylation disorder it may be—dependent on the population—a pragmatic approach to look first for cblC, the most frequent remethylation disorder by searching for the common mutation (c.271dupA) or MMACHC sequencing. If these analyses however prove negative, functional and/or complementation studies in fibroblasts or extended molecular genetic testing for other defects are needed.

For MTHFR deficiency (OMIM 6070993) the majority of mutations are private and neither type nor location of mutation correlates with clinical phenotype (Froese et al 2016). Genetic testing for MTFHR deficiency should be interpreted with caution since there are numerous polymorphisms in this gene, which have been related to various common disorders and conditions. Polymorphisms in general including the most investigated thermolabile variant c.677C>T in MTHFR are not responsible for severe remethylation disorders (Tsang et al 2015).

**Outcome: valid, timely laboratory diagnosis**

Recommendation 6
  We strongly recommend diagnostic confirmation by molecular genetic analysis and/or direct or indirect enzyme assays in cultured skin fibroblasts (or lymphocytes) in experienced laboratories. (Quality of the evidence: moderate)

### Prenatal diagnosis

Metabolites concentration (tHcy and MMA) in cell-free amniotic fluid, enzyme activity of MTHFR and methionine synthase or methionine synthase reductase in cultured amniotic cells and incorporation of propionate and methyltetrahydrofolate have been used for many years (Fowler and Jakobs 1998; Merinero et al 1998) and their combined use is reliable (Morel et al 2005). Indirect enzyme assays in chorion biopsy should be avoided (Morel et al 2005; Merinero et al 1998).

However nowadays, the molecular genetic diagnosis is the most advisable method provided the causal mutations in the index case and carrier status in the parents have been identified. Molecular genetic testing can be performed from chorionic villi or amniotic fluid samples (Morel et al 2005).

**Outcome: valid, timely laboratory diagnosis**

Recommendation 7
  If prenatal diagnosis is considered in individual cases we recommend to perform molecular genetic analysis from chorionic villi or amniotic fluid samples given that mutations in the index case and carrier status in the parents have been identified (Quality of the evidence: low)

### Feasibility and impact on outcome of newborn screening (NBS) for combined remethylation disorders

NBS for the cblC defect should be considered since survival and prevention of severe complications such as HUS, hydrocephalus and haematological abnormalities in early-onset patients can be improved by early treatment (Huemer et al 2015b). The impact of early treatment on neurocognitive development is unclear and early treatment has little influence on eye disease (Weisfeld-Adams et al 2013). In most late-onset cblC patients, dementia, renal function, myelopathy and axonal neuropathy improve on treatment and long-lasting disease existing before treatment initiation correlates with residual pathologies (Huemer et al 2014). There is insufficient data on NBS for other combined remethylation disorders.

**Outcome: survival, severe organ complications; visual and neurocognitive function**

Recommendation 8
  We strongly recommend early treatment in patients with the cblC defect as it improves survival, corrects haematological abnormalities and may prevent HUS and hydrocephalus. However, early treatment has little influence on eye disease and unclear impact on neurocognitive outcome (Quality of the evidence: moderate)

Present knowledge allows no conclusion concerning the clinical benefit of early treatment for the cblD-MMA/HC, cblF and cblJ defects.

The Region 4 Stork (R4S) data indicate that the sensitivity of C3/C2 as primary markers is superior to Met and Met/Phe (McHugh et al 2011). However, specificity of C3 and/or C3/C2 for detecting these diseases is generally low (la Marca et al 2007; Tortorelli et al 2010). The positive predictive value substantially increased from 4 to 100 % by measurement of MMA and 11 to 36 % by measuring tHcy as second tier analytes in DBS (la Marca et al 2007; Tortorelli et al 2010). The sensitivity of C3 and C3/C2 for mild/late-onset forms is unknown. It is of note that neonatal metabolic disturbances due to maternal vitamin B12 deficiency may also be detected. It has recently been shown that heptadecanoyl-carnitine (C17) may have an even higher predictive value for perturbations of MMA metabolism including combined remethylation defects and may thus be a promising first tier analyte (Malvagia et al 2015).

**Outcome: timely and valid laboratory diagnosis**

Recommendation 9
  We strongly recommend to obtain plasma for determination of serum vitamin B12 before treatment is started in cases identified by NBS as part of studies to exclude maternal vitamin B12 deficiency. (Quality of the evidence: moderate)
Recommendation 10
  We recommend use of C3 acylcarnitine and the C3/C2 ratio as primary markers to screen for early onset cblC defect. (Quality of the evidence: moderate)
Recommendation 11
  We suggest consideration of C17 acylcarnitine as a promising primary marker to screen for early onset cblC defect. (Quality of the evidence: low)
Recommendation 12
  We strongly recommend performing second tier testing using tHcy and MMA to improve specificity and to differentiate the defects from other disorders. (Quality of the evidence: moderate)

### Feasibility and impact on outcome of NBS for isolated remethylation disorders and MTHFR deficiency

The use of NBS for detection of the isolated remethylation disorders and the MTHFR defect appears worthy of consideration. In the cblE and cblG defect, macrocytic anaemia (Vilaseca et al 2003; Kvittingen et al 1997) and neurocognitive performance (Harding et al 1997; Rosenblatt et al 1985; Schiff et al 2011; Kvittingen et al 1997; Müller et al 2007) often respond to treatment (Müller et al 2007; Schiff et al 2011). Eye disease seems not to be responsive (Huemer et al 2014). Early detection by NBS and timely treatment improved short-term outcomes of two asymptomatic patients with the cblE and cblG defect and three symptomatic patients with MTHFR deficiency (Wong et al 2016). Moreover, early betaine treatment has a clear positive impact on outcome in MTHFR deficiency (Diekman et al 2014).

Neonatal screening for cblD-HC, cblE and cblG and for MTHFR deficiency appears to be feasible by detecting decreased methionine and methionine-to-phenylalanine ratio (Bowron et al 2005) in DBS. The second-tier marker tHcy clearly differentiates patients from controls (McHugh et al 2011; Tortorelli et al 2010). However, sufficient data on efficacy of NBS programs for these disorders is lacking.

**Outcome: survival, severe organ damage; neurocognitive impairment**

Recommendation 13
  We strongly recommend early identification and treatment with betaine for MTHFR deficiency. Presymptomatic betaine treatment prevents severe neurological impairment (Quality of the evidence: high)

### Disease course and outcome of combined remethylation disorders

Most information on complications and outcome is based on data from patients with cblC disease. There is limited data about the natural history of the other combined disorders (Table 6).

**Table 6:** Main complications according to system in remethylation disorders

Growth and physical features	Prenatal growth retardation and postnatal failure to thrive Dysmorphic facial features
CNS	Microcephaly Hydrocephalus Developmental delay and/or regression; cognitive impairment ranging from executive dysfunction to severe mental retardation Neuropsychiatric disturbances, social withdrawal, personality changes, dementia Progressive encephalopathy Seizures Subacute combined degeneration of the spinal cord Peripheral neuropathy Leukoencephalopathy Cortical atrophy
Eye	Nystagmus Maculopathy Progressive pigmentary retinopathy Optic atrophy Visual impairment/blindness
Blood	(Macrocytic) anaemia Thrombocytopenia and/or neutropenia
Vascular	Stroke Recurrent venous thrombosis Cor pulmonale or subclinical pulmonary thrombosis
Renal	Haemolytic-uremic syndrome Glomerulopathy
Heart	Congenital heart defects Left ventricular non-compaction Dilated cardiomyopathy Pulmonary hypertension

Patients with the cblC defect usually present with severe complications and historically have a poor long-term outcome (Rosenblatt et al 1997; Andersson et al 1999; Fischer et al 2014). It is well recognised that despite treatment and improved metabolic parameters, severe complications such as developmental delay and progressive visual loss may still develop (Andersson et al 1999; Enns et al 1999; Patton et al 2000; Fischer et al 2014; Weisfeld-Adams et al 2015). Even if the neurologic status stabilises or improves with therapy, the sequelae remain in a large proportion of patients (Whitehead 2006; Watkins and Rosenblatt 1989), especially if the initiation of treatment was delayed or insufficient (Huemer et al 2014). Haematological symptoms and failure to thrive generally resolve with treatment (Martinelli et al 2011; Fischer et al 2014).

The late-onset phenotype has a more favourable outcome than early-onset cblC disease but is still associated with residual sequelae such as learning difficulties, neurobehavioural symptoms, neurogenic bladder and gait abnormalities (Rosenblatt et al 1997) and at least one patient, in whom treatment was ceased, deteriorated and died (Thauvin-Robinet et al 2008).

### Mortality

Early detection and treatment with parenteral OHCbl appear to have decreased the mortality in newborns with cblC. A retrospective analysis of 50 patients (44 presented in the first year of life) with cblC disease reported an overall mortality rate of 30 % (Rosenblatt et al 1997). Of the 13 patients that died, four were not treated, two received only cyanocobalamin (CNCbl), and three were initially treated with CNCbl and then switched to OHCbl (Rosenblatt et al 1997; Carrillo-Carrasco et al 2012).

Later, in the Fischer study about outcome in a series of 88 patients with the cblC defect, the mortality was 11.4 % (90 % of them with infantile onset). Treatment following various regimes of parenteral OHCbl complemented in some cases by betaine, folate/folinic acid and carnitine resulted in improvement of biochemical abnormalities, non-neurological signs and mortality (Fischer et al 2014). It has been suggested that daily treatment with parenteral OH-Cbl combined with betaine would be associated with a favourable outcome (Carrillo-Carrasco et al 2012).

### Renal disease and microangiopathy

Thrombotic microangiopathy (TMA) leading to atypical HUS is the most common renal manifestation in cblC; it has also been observed in cblD-MMA/HC patients. HUS may cause hypertension, intravascular haemolysis, microscopic haematuria, proteinuria and renal function deterioration, occasionally proceeding to renal failure (Van Hove et al 2002; Guigonis et al 2005; Sharma et al 2007; Geraghty et al 1992; Morath et al 2013). A case of focal segmental glomerulosclerosis and atypical glomerulopathy (Brunelli et al 2002) has been reported. Histological findings of the kidneys include widening of the mesangium, swelling of endothelial cells with detachment from the basement membrane, and granular deposits in the subendothelial space (Van Hove et al 2002; Russo et al 1992; Brunelli et al 2002; McCully 1969). Isolated pulmonary hypertension has been observed (Iodice et al 2013; Gündüz et al 2014) in cblC patients. Early onset combined pulmonary hypertension and renal thrombotic microangiopathy with a fatal course in several untreated cases has been reported (Kömhoff et al 2013), this picture is also seen in rare cases of late onset cblC patients (Grangé et al 2015). Timely treatment can improve outcome significantly (Grangé et al 2015; Kömhoff et al 2013; Gündüz et al 2014).

**Outcome: survival, severe organ complications**

Recommendation 14
  We recommend monitoring for all aspects of renal disease including arterial blood pressure in patients with cobalamin related remethylation disorders. (Quality of the evidence: low)

### Vascular problems

Thromboembolic complications are an important cause for morbidity and mortality in patients with cblC disease (Martinelli et al 2011; Thauvin-Robinet et al 2008). These include recurrent venous thrombosis (Thauvin-Robinet et al 2008; Roze et al 2003; Augoustides-Savvopoulou et al 1999; Bodamer et al 2001; Powers et al 2001; Guigonis et al 2005), pulmonary thrombosis (Powers et al 2001; Thauvin-Robinet et al 2008; McCully 1969; Baumgartner et al 1979a, b; Van Hove et al 2002), cor pulmonale (Profitlich et al 2009a, b) and cerebrovascular complications (Brunelli et al 2002; Geraghty et al 1992).

tHcy levels above 45 μmol/L have been reported to be associated with the development of vascular complications in several patients (Carrillo-Carrasco et al 2009). Remarkably, the original case of cblC disease presenting diffuse vascular lesions with proliferative fibrous intimal plaques and focal necrosis of the artery wall contributed to the development of the homocysteine theory of arteriosclerosis (McCully 1992).

With appropriate treatment, the incidence of thromboembolic complications can be reduced and may even be prevented in late onset patients (Huemer et al 2014).

**Outcome: survival, severe organ complications**

Recommendation 15
  The incidence of vascular complications is significantly reduced with appropriate treatment in late onset patients and may be prevented in early onset patients with remethylation disorders. (Quality of the evidence: moderate)

### Neurocognitive and psychiatric problems

Neurodevelopmental impairment of varying degrees is common in combined remethylation defects. Patients with early-onset cblC disease present with a wide range of neurological manifestations that include microcephaly, hydrocephalus, hypotonia, cognitive defects and seizures. Cognitive defects can be variable, are related to disease severity and treatment onset but may also continue to worsen despite treatment. Two treated patients with early-onset cblC disease were longitudinally followed using neuropsychological testing. These tests showed a decline in attention and executive functions, while other skills were relatively spared (Beauchamp et al 2009). Developmental delay with impairment of verbal and non-verbal cognitive skills was also documented at follow-up of cblF patients despite treatment (Alfadhel et al 2011; Gailus et al 2010), but in some early treated patients, neurocognitive outcome was satisfactory (Miousse et al 2011; Armour et al 2013). Information on outcome in the cblJ defect (Coelho et al 2012) is extremely limited; two reported patients responded well to methylcobalamin (Kim et al 2012). In the cblD-MMA/HC defect, some survivors showed improvement or even normalisation of neurocognitive function (Suormala et al 2004; Miousse et al 2009).

Patients with late-onset disease can show progressive encephalopathy with regression, deterioration in school or work performance, behavioural and personality changes possibly resulting in dementia, psychosis, episodes of acute mental confusion, lethargy and seizures that improve under specific treatment (Huemer et al 2014).

Epilepsy is a common but nonspecific occurrence in patients with remethylation disorders. The EEG and seizure patterns are nonspecific. Severely affected patients may be difficult to treat and may have recurrent status epilepticus (Biancheri et al 2002).

Brain MRI abnormalities are common in remethylation defects and include diffuse cerebral atrophy, white matter changes, basal ganglia lesions and hydrocephalus.

The most common imaging findings in the early-onset cblC cases are variable degrees of white matter abnormality with T2 hyperintensity in periatrial and periventricular white matter, with thinning of the corpus callosum. Basal ganglia lesions and hydrocephalus have also been described, and may be attributable to microvascular abnormalities (Greitz 2007). In one cohort of young early-onset cblC patients, there was a high incidence of craniocaudal shortening of the pons on sagittal MR images (Weisfeld-Adams et al 2013).

Cerebral atrophy and deep white matter bulk loss, which is more pronounced posteriorly, were significant findings in late-onset cblC disease patients (Ogier de Baulny et al 1998; Longo et al 2005; Rossi et al 2001; Wang et al 2012). Bilateral abnormalities of the cerebellar cortex were found in one patient (Wang et al 2012).

The pathophysiological mechanisms underlying the white matter abnormalities detected by MR imaging have been associated with oedema and abnormal myelination. Myelination is closely related to methylation capacity whereby deficiency of SAM in the CSF was shown to be associated with impaired myelinisation (Rossi et al 2001). Moreover, a decrease in N-acetyl aspartate (NAA) and increased lactate in the basal ganglia or in the periventricular white matter has been observed on brain MR spectroscopy (Longo et al 2005; Wang et al 2012).

Spongiform white matter degeneration and demyelination of the dorsal and lateral columns of the cord or subacute combined degeneration of the spinal cord (SACD) are known complications in remethylation disorders (Huemer et al 2014; Liu et al 2015). Changes are similar to classical adult spinal cord degeneration due to, e.g. severe nutritional vitamin B12 deficiency (Scalabrino et al 2007; Maamar et al 2008). Subacute combined degeneration of the spinal cord may be the presenting symptom in late onset patients or the consequence of insufficient treatment. Symptoms are progressive and include numbness of lower extremities, gait disturbances, incontinence, progressive leg weakness with spastic paraparesis and tetraplegia (Mitchell et al 1986; Ben-Omran et al 2007; Thauvin-Robinet et al 2008; Augoustides-Savvopoulou et al 1999; Powers et al 2001; Roze et al 2003; Tsai et al 2007; Bodamer et al 2001; Shinnar and Singer 1984).

Behavioural problems are frequent in patients with early-onset remethylation disorders. Psychiatric manifestations may be the presenting sign in patients with late-onset remethylation disorders. Dementia, behavioural problems and psychiatric symptoms have frequently been observed in the combined (Roze et al 2003; Liu et al 2015; Huemer et al 2014) as well in isolated remethylation disorders (Huemer et al 2015a) and MTHFR deficiency (Birnbaum et al 2008). In early onset cases, behavioural problems and psychiatric symptoms can evolve over time despite treatment (Fischer et al 2014). Late-onset cases may present with rather non-specific psychiatric and behavioural symptoms, which respond well to timely treatment (Ben-Omran et al 2007).

### Ophthalmological problems

Visual dysfunction is very frequent in patients with early onset remethylation disorders and can be rapidly progressive while late-onset patients rarely exhibit eye disease. Current treatment does not seem to modify the progression of visual disease significantly.

Maculopathy, progressive retinal dysfunction, visual impairment, nystagmus, strabismus (Ricci et al 2005; Schimel and Mets 2006; Gerth et al 2008; Weisfeld-Adams et al 2015) and less frequently optic atrophy (Patton et al 2000; Weisfeld-Adams et al 2015) are seen in most patients with early onset cblC disease. Although rod cell sensitivity was reported to improve in a patient with cblC disease following normalisation of plasma Met levels (Tsina et al 2005) generally retinal dysfunction can be progressive and even lead to blindness despite treatment and stabilised systemic function (Fischer et al 2014).

The first signs of eye disease are frequently noted several weeks after birth and include “wandering eye movements,” inability to fixate and nystagmus. The fundoscopic exam can reveal the salt-and-pepper pigmentary macular changes that progresses to the characteristic “bull’s eye” maculopathy characterised by a hypopigmented perimacular zone surrounded by a hyperpigmented ring or coloboma like lesions (Robb et al 1984; Grant et al 2009; Weisfeld-Adams et al 2015). Retinopathy can be revealed by the electroretinogram well before clinical signs are apparent (Ogier de Baulny et al 1998; Weisfeld-Adams et al 2015) showing a progressive decline in scotopic (cones) and/or photopic (rods) responses (Schimel and Mets 2006; Robb et al 1984; Gerth et al 2008; Gaillard et al 2008; Weisfeld-Adams et al 2015).

Ocular coherence tomography, electroretinogram and visual evoked potentials, are extremely useful diagnostic and monitoring tools (Weisfeld-Adams et al 2015). Knowledge and awareness of visual dysfunction, particularly in children with early-onset disease, allows initiation of appropriate early vision intervention programs and support (Gerth et al 2008).

Late onset patients rarely have severe visual disease (Fuchs et al 2012; Fischer et al 2014; Gerth et al 2008). Minimal pigmentary retinal abnormalities have been observed in some cases (Gerth et al 2008; Weisfeld-Adams et al 2015) and the classical “bull’s eye” picture has only been described in one late-onset patient (Collison et al 2015).

**Outcome: visual and neurocognitive function**

Recommendation 16
  As knowledge and awareness of visual dysfunction progression allows timely initiation of appropriate vision intervention programs and support, we recommend that every patient newly diagnosed with a remethylation disorder should receive an ophthalmological consultation independent of the age at diagnosis and severity of disease. (Quality of the evidence: low)

## Disease course and outcome in isolated remethylation disorders (cblD-HC, cblE, cblG)

Disease course and outcome in isolated remethylation disorders generally share many features with the combined disorders.

### Mortality

Deceased patients have been reported in the literature but the database is too small to make a more general statement on mortality.

### Renal disease, microangiopathy and anaemia

Atypical HUS and glomerulopathy have been reported in single patients (Paul et al 2013; Huemer et al 2015a). Macrocytic anaemia in isolated remethylation defects often responds to treatment (Harding et al 1997; Vilaseca et al 2003).

### Vascular problems

Thromboembolism may lead to severe clinical pathology but has only rarely been reported (Huemer et al 2015a).

### Neurocognitive and psychiatric problems

Muscular hypotonia, seizures, cognitive impairment, behavioural problems, psychiatric symptoms, feeding problems (Watkins and Rosenblatt 1989; Huemer et al 2015a) as well as microcephaly, brain atrophy and white matter changes (Zavadáková et al 2005) are characteristic findings during the disease course (Watkins and Rosenblatt 1989). Hydrocephalus has rarely been reported (Huemer et al 2015a).

### Ophthalmological problems

Eye disease, mainly comprising retinopathy, nystagmus and strabismus is frequently observed and seems not to respond well to treatment (Watkins and Rosenblatt 1989; Huemer et al 2015a).

## Disease course and outcome in MTHFR deficiency

### Mortality

Recent experience suggests that betaine treatment if initiated early reduces mortality in patients with MTHFR deficiency (Diekman et al 2014)

### Vascular problems

Arterial and venous thrombosis are generally rare and predominantly encountered in adolescent or adult patients (Visy et al 1991; Tonetti et al 2002).

### Neurocognitive problems

Severe neurological signs with some age-related variations characterise MTHFR deficiency. Neurological symptoms in MTHFR range from seizures, lethargy, apnoea and coma in early infancy to gait disturbances with lower limb spasticity, weakness, psychiatric behaviour such as schizophrenia-like psychosis (Mudd and Freeman 1974; Regland et al 1994; Pasquier et al 1994), and visual problems in adolescence/adulthood (Haworth et al 1993; Birnbaum et al 2008). Neonates and young infants exhibit acute neurological deterioration, which may be fatal or leave severe neurological deficits. Older individuals typically exhibit progressive triphasic deterioration: an initial period of normal development, then a second phase with acquired microcephaly and psychomotor delay, followed by abrupt deterioration associated with respiratory failure that may be fatal.

In early onset severe forms clinical sequelae associated with MTHFR deficiency include microcephaly, hydrocephalus, seizures, hypotonia and global development delay, whereas in the adolescence onset form mental retardation and progressive encephalopathy are present (Arn et al 1998; Bishop et al 2008; Schiff et al 2011).

Some patients develop leukoencephalopathy and lower limb-dominant demyelinating polyneuropathy (Walk et al 1994) and may have subacute degeneration of the spinal cord (Hyland et al 1988). The peripheral neuropathy has been related to the low level of serum folic acid (Nishimura et al 1985). Seizures are variable in type and include myoclonic, clonic and/or tonic episodes. Infantile spasms have been described (Prasad et al 2011). Cerebral atrophy and white matter changes are common (Arn et al 1998; Birnbaum et al 2008).

Early treated patients exhibit a more favourable outcome (Schiff et al 2011; Diekman et al 2014).

In MTHFR deficiency, early diagnosis and treatment are usually associated with better neurological outcome.

## The effects of treatment on clinical outcome

### Impact of prenatal treatment on outcome

Prenatal maternal treatment with parenteral OHCbl has anecdotally been used. For cblC disease, Trefz et al (2016) report a favourable outcome of a pregnancy with an affected fetus (sibling to a severely affected child) after treatment of the mother with high-dose OHCbl (30 mg/week) and 5 mg/day folic acid from week 15 of pregnancy. Prenatal treatment with lower OHCbl doses resulted in biochemical improvement. Organ complications such as microangiopathy as present in the patient’s sibling were not observed but the patient developed eye disease and neurocognitive impairment (Huemer et al 2005). There is no evidence regarding prenatal treatment for the other diseases.

**Outcome: survival, severe organ damage**

Recommendation 17
  We suggest that prenatal maternal treatment may be considered in a pregnancy with a fetus with proven cblC disease. (Quality of the evidence: low)

### Improving the outcome in the acutely ill patient with a suspected or proven remethylation disorder

Remethylation disorders generally present as chronic, slowly progressive neurological disease but may also present with acute deterioration on the background of chronic disease or with new-onset life-threatening complications. Individuals of all ages may manifest with a sudden thromboembolic event, microangiopathic kidney or pulmonary disease, cardiomyopathy, loss of ambulation or more commonly with acute encephalopathy, displaying epileptic seizures impaired consciousness or acute behavioural deterioration. Restoration of methylation capacity with appropriate medication may reverse many of the acute clinical signs (Fischer et al 2014; Huemer et al 2014) (Fig. 2).

**Outcome: survival, severe organ damage; neurocognitive impairment**

Recommendation 18
  We strongly recommend immediate treatment with parenteral cobalamin in suspected cases. (Quality of the evidence: moderate)
Recommendation 19
  We recommend consideration of betaine treatment as soon as hyperhomocysteinaemia is proven and normal/low methionine confirmed. (Quality of the evidence: moderate)
Recommendation 20
  We suggest consideration of additional enteral supplementation with folinic acid or L-methionine in individual cases. (Quality of the evidence: low)

### Improving the outcome: long-term management of cobalamin-related combined and isolated remethylation disorders

Most of the knowledge about the management of remethylation disorders is derived from the experience with individuals with the cblC defect, the most frequent of the disorders. In clinical practice the rarer combined disorders of the cblD-MMA/HC, cblF and cblJ types as well as the isolated remethylation disorders are generally managed identically but experience concerning treatment efficacy is considerably less and weaker.

Treatment of individuals with the remethylation disorders aims to improve clinical features and metabolic abnormalities by reducing tHcy and normalising Met and—in combined disorders—MMA (Table 7).

**Table 7:** Recommended treatment of remethylation disorders

	Drugs with proven clinical effect	Treatments without proven clinical effect	To be avoided
Cobalamin related remethylation disorders	OHCbl parenteral Betaine	Folate/folinic acid L-Carnitine Methionine*	Nitrous oxide Protein restriction
MTHFR deficiency	Betaine	Folinic acid/ 5-Methylfolate* L-Carnitine Methionine*	Nitrous oxide Folic acid Protein restriction

^*^Has been applied with clinical benefit in single cases

#### Cobalamin

Cobalamin is the cofactor of methionine synthase. In the cblC defect, hydroxocobalamin (OHCbl) appears to be more effective than cyanocobalamin (CNCbl) (Andersson and Shapira 1998; Bodamer et al 2001). Froese et al (2010) describe that, e.g. MMAMHC with the c.482G>A (R161Q) mutation binds OHCbl with more affinity than CNCbL. The parental route of administration (IV, SQ or IM) has been proved to be beneficial in patients with the cblC defect (Van Hove et al 2002), whereas oral OHCbl alone seems to be ineffective (Brunelli et al 2002; Bartholomew et al 1988; Gold et al 1996; Frattini et al 2010). Long-term IV application is impracticable and the subcutaneous route seems to be less effective than IM injections (Thauvin-Robinet et al 2008). Many centres use 1 mg of parenteral OHCbl daily in neonates (assuming a body weight of 3 kg, the dosage is 0.33 mg/kg/day). In the long-term treatment, as patients stabilise, the frequency of administration is decreased to minimise the frequency of injections. However, determination of optimal doses and intervals between injections is hampered by very great variation in patient features (Dionisi-Vici et al 2013; Ogier de Baulny et al 1998). Some recent reports suggest that higher OHCbl doses (plasma levels near 1,000,000 pg/ml) can provide a better metabolic control and dose escalation seems to be effective in several reports, including a case of pregnancy in a cblC affected female (Brunel-Guitton et al 2010). In 2002 Van Hove et al (2002) described a good response to OHCbl dose escalation regarding metabolic and clinical condition in two cblC patients presenting with thrombotic microangiopathies (TMAs). During 13 years of follow up in a cblC patient Carrillo-Carrasco et al (2009) observed a considerable dose-dependent reduction of MMA and tHcy plasma levels and an elevation of methionine levels as the dose of IM OHCbl was increased. Matos et al 2013 performed an open-label study in five cblC patients to determine the effect of OHCbl dose escalation on the clinical and biochemical condition. They observed a good metabolic response in 2/5 patients, whereas clinical response was quite variable and not clearly related to metabolic improvement. In early onset cblC disease, tHcy mostly cannot be normalised; levels between 40 and 60 μmol/L are typically reached. In some late onset patients, tHcy has been normalised.

There is limited experience with OHCbl (1000 μg/day) treatment in cblD-MMA/HC disease but there seems to be a response to treatment (Miousse et al 2009; Suormala et al 2004). Most patients with cblE and cblG disease were treated with OH-Cbl and seemed to benefit (Huemer et al 2015a, b). As OH-Cbl is generally used to treat other cobalamin-related remethylation disorders than cblC disease, no sufficient data is available on the clinical efficacy of other cobalamin preparations .

In conclusion, OHCbl treatment is usually started at a dosage of 1 mg IM daily and then it should be titrated individually based on metabolic response.

**Outcome: survival and severe organ damage**

Recommendation 21
  We strongly recommend using parenteral OHCbl in treating patients with the cblC defect and other cobalamin-related remethylation disorders. (Quality of the evidence: high)
Recommendation 22
  We recommend applying a starting dose of 1000 μg (1 mg) OHCbl daily given parenterally in patients with the cblC defect. This regime has also been applied in other cobalamin-related remethylation defects. (Quality of the evidence: low)
Recommendation 23
  We suggest that the minimum effective OHCbl dose and frequency of administration should be individually titrated. Escalating doses of OHCbl may result in biochemical improvement; however, significant clinical benefit remains to be proven. Frequency of administration of OHCbl ranges between daily and weekly without evidence of advantage of one over the other. (Quality of the evidence: low)

#### Betaine

Betaine derives from choline and is a potent methyl group donor involved in the process of remethylation of Hcy to Met via betaine-homocysteine methyltransferase in the liver, thus bypassing the MeCbl-dependent pathway. Betaine needs to be supplemented orally and decreases Hcy levels. The knowledge about betaine pharmacokinetics is limited (Schwahn et al 2003). It has been suggested that frequent administration of a moderate dose may provide clinical and biochemical benefit (Ucar et al 2010). Betaine or OHCbl alone may not result in sufficient metabolic control, but they seem to have synergistic effects (Bartholomew et al 1988). Betaine is usually administered at a dose of up to 250 mg/kg/day in children (Beauchamp et al 2009; Schiff et al 2011), but has been used at a dose of 850 mg/kg/day to treat cor pulmonale in a patient with the cblC defect (Profitlich et al 2009a, b). Betaine has also been used during pregnancy with no neonatal adverse effect (Pierre et al 2006). Anhydrous betaine is the preferred formulation.

**Outcome: survival and severe organ damage**

Recommendation 24
  We recommend oral betaine treatment in cblC disease and other cobalamin-related remethylation disorders (Quality of the evidence: low)
Recommendation 25
  We suggest that the minimum effective betaine dose should be individually titrated to improve the levels of tHcy and methionine. (Quality of the evidence: low)

#### Side effects of treatment with cobalamin and betaine

Few side effects related to cblC disease treatment have been reported. These are usually scarce and reversible as soon as the treatment is withdrawn.

OHCbl side effects such as red discoloration of the urine and skin, hypersensitivity and photosensitivity reactions, nausea, infusion site reactions and headache have rarely been reported in patients receiving high doses. Mostly, parenteral OHCbl even in doses as high as 20 mg per day have been tolerated without side effects (Carrillo-Carrasco et al 2009).

In a single case, teeth discoloration occurred after 1 month of OHCbl (1 mg/day IM) and folinic acid (10 mg/day) treatment and improved after folinic acid discontinuation (Augoustides-Savvopoulou et al 1999).

Long-tem follow up case series and case reports indicate that betaine is generally well tolerated and safe even at high doses (3–6 g/day in infants; 9 g/day in adults) (Ogier De Baulny et al 1998; Thauvin-Robinet et al 2008). However, in a 15-month-old, epileptic crises have been observed soon after initiation of betaine treatment (250 mg/kg/day) (Enns et al 1999).

Betaine decreases Hcy levels at the expense of increasing Met. The clinical relevance of high Met is under discussion but very high levels have been related to cerebral oedema. In a 10-year-old female with classical homocystinuria plasma Met increased to >3000 μmoL/L under betaine treatment (200 mg/kg/day). The girl developed clinical signs and radiological evidence of cerebral oedema which resolved when betaine was discontinued and Met levels returned to normal (Yaghmai et al 2002). Devlin et al (2004) reported on a similar clinical course in a boy at a plasma Met level of 1190 μmol/L. Therefore, caution is required and betaine should immediately be withdrawn in any case of any symptoms suggesting increased intracranial pressure or very high Met levels (Lawson-Yuen and Levy 2006).

#### Folates

Folate has been frequently used as adjunctive therapy in patients with cblC disease. Folinic acid (5-formyl-THF) is the most stable form of the reduced vitamin and crosses the blood brain barrier more efficiently than folic acid (Spector 1979). Daily doses from 5 to 30 mg, divided in 2–3 administrations/week of folate or folinic acid (Carrillo-Carrasco et al 2012; Schiff et al 2011) have been used. In the majority of reported patients, folic acid rather than folinic acid has been used in the long-term treatment. In some cases no beneficial effect of the adjunctive therapy with folic or folinic acid has been reported (Enns et al 1999; Bartholomew et al 1988; Fischer et al 2014) but no long-term studies have been performed.

Recommendation 26
  Results failed to demonstrate or exclude a beneficial or detrimental effect of folic and/or folinic acid as adjunctive therapy in patients with cblC disease and other cobalamin-related remethylation disorders. (Quality of the evidence: low)

#### Levocarnitine

Levocarnitine facilitates the excretion of propionyl groups and prevents carnitine deficiency, since the *de novo* synthesis of carnitine depends on methionine and may be decreased in cblC patients. Some reports in literature show no beneficial effect of adding carnitine to the long-term treatment of cblC disease (Enns et al 1999; Bartholomew et al 1988). There is no clear consensus about the recommended dose and this can range between 50 and 200 mg/kg/day.

Recommendation 27
  Results failed to demonstrate or exclude a beneficial or detrimental effect of oral carnitine as adjunctive therapy in patients with cblC disease and other cobalamin-related remethylation disorders. (Quality of the evidence: low)

#### Dietary restrictions

Few reports describe the use of dietary restriction in cblC patients, mainly with no beneficial effect on metabolic control (Carrillo-Carrasco et al 2009; Enns et al 1999). Huemer et al (2005) report significantly lower MMA excretion upon protein restriction (methionine-free, threonine-free, valine-free, isoleucine-low formula). However, there is evidence suggesting a possible role of low methionine as a contributing factor for the pathogenesis of cblC disease (Martinelli et al 2011) and other remethylation disorders. Met is essential in patients with remethylation defects and its plasma levels should be maintained in the normal range. Patients on protein-restricted diets had lower height-for-age z-score. Patients consuming medical foods with presumably low Met supply had lower head circumference and lower plasma Met concentrations (Manoli et al 2015). For these reasons, methionine-free formulas and protein restriction leading to low methionine plasma levels is contraindicated in cblC patients (Ogier de Baulny et al 1998; Manoli et al 2015).

**Outcome: survival, severe organ damage; neurocognitive impairment**

Recommendation 28
  We strongly recommend not to restrict protein in cblC disease and other remethylation disorders. (Quality of the evidence: moderate)

#### Amino acid supplementation

In patients with cblC defects methionine and cysteine supplements are mostly not required to maintain normal plasma levels of these two amino acids and in a recent retrospective multicentre study evaluating clinical, biochemical and therapeutic measures in 88 cblC patients (Fischer et al 2014) supplemental Met was used only in one patient; cysteine supplementation was never applied. Some patients were treated with betaine and Met in combination when plasma Met was low (Smith et al 2006).

**Outcome: survival, severe organ damage; neurocognitive impairment**

Recommendation 29
  Met is essential in patients with remethylation defects and we recommend maintaining its plasma levels in the normal range; if necessary this may be achieved by oral methionine supplementation. (Quality of the evidence: low)

**Improving the outcome: long-term management of MTHFR deficiency**

#### Betaine

In MTHFR deficiency betaine in a variable dose of 100–250 mg/kg/day in children and 5–20 g/day in adults is used to increase systemic Met levels. A meta-analysis of case reports (Diekman et al 2014) revealed that betaine treatment at a dosage of 100 mg/kg/day or more was clearly associated with increased survival. Only two of 26 patients died after initiation of treatment. Furthermore, early treatment was associated with normal psychomotor development in all five patients. In contrast, none of the 19 patients in the delayed-treatment group (untreated patients excluded) had normal psychomotor development. Nevertheless, psychomotor development stabilised or improved in all 17 surviving patients in whom betaine treatment was initiated late. Anhydrous betaine is the preferred formulation.

**Outcome: survival, severe organ damage; neurocognitive impairment**

Recommendation 30
  We strongly recommend early treatment with betaine as it improves clinical outcome and prevents neurological deterioration in MTHFR deficiency. (Quality of the evidence: high)

*See also recommendation 13*

#### Folates

In MTHFR deficiency 5-methylTHF synthesis is impaired, resulting in low levels of methyl-tetrahydrofolate (CH3-THF) in the central nervous system. There have been some reports describing the use of folic acid (5–80 mg/day) with good (Al Tawari et al 2002; Munoz et al 2015) or poor (Holme and Ronge 1989; Ucar et al 2010) response. A dose–response relationship was not observed (Holme and Ronge 1989). A combination of folic and folinic acid was associated with favourable outcome (Tallur et al 2005; Lossos et al 2014). It has recently been shown that folic acid may exacerbate cerebral CH3-THF deficiency (Hyland et al 2010) and it has therefore been suggested to avoid folic acid and preferably use folinic acid or 5-CH3-THF. However, therapeutic 5-CH3-THF in a daily dose as high as 45 mg failed to correct the low CSF levels in a patient with MTHFR deficiency (Knowles et al 2016; Schiff and Blom 2012). There is at present no consensus concerning the most effective dosage of folinic acid or 5-CH3-THF and the problem of the assumed instability of 5-CH3-THF has not yet been solved.

**Outcome: neurocognitive impairment**

Recommendation 31
  Results failed to demonstrate or exclude a beneficial or detrimental effect of folic or folinic acid or 5-CH3THF as adjunctive therapy to restore cellular and cerebral folate deficiency in MTHFR deficiency on clinical outcome. (Quality of the evidence: low).

#### Other substances

The data basis addressing the efficacy of vitamin B6, cobalamin, riboflavin and carnitine is not reliable; various dosages and combinations of these substances have been used and their clinical efficacy cannot be assessed (Huemer et al 2016).

#### Methionine supplementation

In a very small number of patients, the clinical condition improved with supplementation of methionine (Schiff et al 2011; Tortorelli et al 2010), however, the data basis is not reliable enough to allow delineation of a recommendation.

### Improving clinical outcome: management of general anaesthesia in remethylation disorders

A fatal adverse event was reported in a patient with MTHFR deficiency anaesthetised with nitrous oxide (N2O) which is a known inhibitor of methionine synthase (Selzer et al 2003; Erbe and Salis 2003). In seven patients with the cblC and one with the cblG defect, propofol was safe and effective as an induction and maintenance agent for elective short procedures in metabolically and hemodynamically stable patients (Ktena et al 2015).

Recommendation 32
  We strongly recommend against the use of nitrous oxide in patients with remethylation disorders. (Quality of the evidence: high)

### Improving clinical outcome: management of pregnancy in remethylation disorders

There are only three reported cases of pregnancies in women with the cblC defect and thus no recommendations can be delineated. An asymptomatic woman was identified when low carnitine levels were found in her healthy child’s newborn screening sample (Lin et al 2009). Brunel-Guitton et al (2010) describe successful outcome of a pregnancy in a treated woman with the cblC defect. Acetylsalicylic acid at the antiplatelet oral dose of 80 mg/day was applied during pregnancy and delivery and specific treatment with OHCbl, folic acid and carnitine was started at 15 weeks of gestation. The OHCbl and carnitine administration was adjusted to maintain a decrease of tHcy and an increase of total carnitine plasma levels. Recently a woman with late-onset cblC, also treated from the 15th week of gestation and giving birth to a healthy child has been reported (Liu et al 2015). So far, pregnancies in women with other remethylation defects have not been reported.

## Closing remarks

These guidelines are the result of the evaluation of available information from the literature based on the GRADE methodology and are aimed at delivering recommendations based on the best available data. The rarity of cobalamin-related remethylation disorders and MTHFR deficiency, the absence of larger data samples from international registries until recently and data mainly derived from case reports, case series or expert opinion results in a low quality of the evidence for several of the recommendations made. The working group responsible for this guideline commits itself to revise this work in the future.

## Electronic supplementary material

Below is the link to the electronic supplementary material.

ESM 1 (DOCX 32 kb)
