# Supplementary material for: Guidelines for diagnosis and management of the cobalamin-related remethylation disorders cblC, cblD, cblE, cblF, cblG, cblJ and MTHFR deficiency
Source: J Inherit Metab Dis. 2016 Nov 30;40(1):21–48. doi: 10.1007/s10545-016-9991-4 (PMC5203859; doi:10.1007/s10545-016-9991-4)
Supplement: Supplementary file 1 — (DOCX 32 kb) [file 10545_2016_9991_MOESM1_ESM.docx]

**Supplementary table 1: quality of the evidence summary**

**Outcome: timely clinical diagnosis**

1. We strongly recommend consideration of an acquired or genetic disorder of remethylation in case of neurological and/or visual and/or haematological symptoms. (Quality of the evidence: moderate)

| Grade of recommendation | Benefit vs risk and burdens | Strength of supporting evidence |  |
| --- | --- | --- | --- |
| Strong (87%) | Benefits clearly outweigh risks & burdens | Moderate  (upgraded for high consistency) | 174 clinical cases / case series |

1. We strongly recommend considering an acquired or genetic disorder of remethylation in case of unexplained thrombosis and / or spinal cord degeneration and/or atypical HUS. (Quality of the evidence: moderate)

| Grade of  recommendation | Benefit vs risk and burdens | Strength of supporting  evidence |  |
| --- | --- | --- | --- |
| Strong (87%) | Benefits clearly outweigh risks & burdens | Moderate  (upgraded for high con-sistency) | 174 clinical cases / case series |

**Outcome: valid, timely laboratory diagnosis**

1. Elevated plasma tHcy is the hallmark of remethylation disorders. We strongly recommend that investigations in patients with a suspected remethylation disorder should start with the measurement of total homocysteine in blood. We recommend the blood sample for tHcy to be centrifuged within an hour and kept at +4° or frozen until analysis. Immunoassays or chromatographic methods are suitable for tHcy measurement. (Quality of the evidence: moderate)

| Grade of  recommendation | Benefit vs  risk and burdens | Strength of  supporting evidence |  |
| --- | --- | --- | --- |
| Strong  (100%) | Benefits clearly outweigh risks & burdens | Moderate  (upgraded for high consistency) | 174 clinical cases / case series; 5 publications on methods |

1. We strongly recommend against measuring free homocysteine instead of total homocysteine. (Quality of the evidence: moderate)

| Grade of  recommendation | Benefit vs  risk and burdens | Strength of  supporting evidence |  |
| --- | --- | --- | --- |
| Strong (100%) | Benefits clearly outweigh risks & burdens | Moderate  (upgraded for high consistency) | 3 biochemical publications, textbook knowledge on homocysteine and homocystine |

1. We strongly recommend that in case of high total homocysteine, plasma and urine samples for determination of MMA, methionine, folate and vitamin B12 are to be obtained before treatment is started. (Quality of the evidence: moderate)

| Grade of  recommendation | Benefit vs  risk and burdens | Strength of  supporting evidence |  |
| --- | --- | --- | --- |
| Strong (93%) | Benefits clearly outweigh risks & burdens | Moderate  (upgraded for high consistency) | 174 clinical cases / case series publications  Basic publications, general reviews and textbook knowledge of Vitamin B12 deficiency |

**Outcome: valid, timely laboratory diagnosis**

1. We strongly recommend diagnostic confirmation by molecular genetic analysis and / or direct or indirect enzyme assays in cultured skin fibroblasts (or lymphocytes) in experienced laboratories. (Quality of the evidence: moderate)

| Grade of  recommendation | Benefit vs  risk and burdens | Strength of  supporting evidence |  |
| --- | --- | --- | --- |
| Strong (93%) | Benefits clearly outweigh risks & burdens | Moderate  (upgraded for high consistency) | 174 clinical cases / case series |

1. If prenatal diagnosis is considered in individual cases we recommend to perform molecular genetic analysis from chorionic villi or amniotic fluid samples given that mutations in the index case and carrier status in the parents have been identified (Quality of the evidence: low)

| Grade of  recommendation | Benefit vs  risk and burdens | Strength of  supporting evidence |  | Comment |
| --- | --- | --- | --- | --- |
| Strong (100%) | Benefits clearly outweigh risks & burdens | Low | 2 publications on specific diagnostics; textbook knowledge of prenatal diagnosis | No absolute recommendation due to low strength of evidence |

**Outcome: survival, severe organ complications; visual and neurocognitive function**

1. We strongly recommend early treatment in patients with the cblC defect as it improves survival, corrects haematological abnormalities and may prevent HUS and hydrocephalus. However, early treatment has little influence on eye disease and unclear impact on neurocognitive outcome (Quality of the evidence: moderate)

| Grade of  recommendation | Benefit vs  risk and burdens | Strength of  supporting evidence |  |
| --- | --- | --- | --- |
| Strong (70%) | Benefits clearly outweigh risks & burdens | Moderate  (upgraded for high consistency) | 98 case reports / case series |

**Outcome: timely and valid laboratory diagnosis**

1. We strongly recommend to obtain plasma for determination of serum vitamin B12 before treatment is started in cases identified by NBS as part of studies to exclude maternal vitamin B12 deficiency. (Quality of the evidence: moderate)

| Grade of  recommendation | Benefit vs  risk and burdens | Strength of  supporting evidence |  |
| --- | --- | --- | --- |
| Strong (100%) | Benefits clearly outweigh risks & burdens | Moderate  (upgraded for high consistency) | 174 clinical cases / case serie  Basic publications, general reviews and text-book knowledge of Vitamin B12 deficiency |

1. We recommend use of C3 acylcarnitine and the C3/C2 ratio as primary markers to screen for early onset cblC defect. (Quality of the evidence: moderate)

| Grade of  recommendation | Benefit vs  risk and burdens | Strength of  supporting evidence |  | Comment |
| --- | --- | --- | --- | --- |
| Strong (64%) | Benefits clearly  outweigh risks &  burdens | Moderate  (upgraded for  high consistency) | Review on NBS +  6 NBS publications | Completely different strategies (e.g. tHcy 1^st^ tier) generally possible, therefore no absolute recommendation |

1. We suggest consideration of C17 acylcarnitine as a promising primary marker to screen for early onset cblC defect. (Quality of the evidence: low)

| Grade of  recommendation | Benefit vs  risk and burdens | Strength of  supporting evidence |  | Comment |
| --- | --- | --- | --- | --- |
| Weak (47%) | Uncertainty in the estimates of  risks & burdens | Low | 2 publications | Data extremely scarce and from single centre |

1. We strongly recommend performing second tier testing using tHcy and MMA to improve specificity and to differentiate the defects from other disorders. (Quality of the evidence: moderate)

| Grade of  recommendation | Benefit vs  risk and burdens | Strength of  supporting evidence |  |
| --- | --- | --- | --- |
| Strong (93%) | Benefits clearly outweigh risks & burdens | Moderate  (upgraded for high consistency) | Review on NBS +  6 additional NBS publications |

**Outcome: survival, severe organ damage; neurocognitive impairment**

1. We strongly recommend early identification and treatment with betaine for MTHFR deficiency. Presymptomatic betaine treatment prevents severe neurological impairment (Quality of the evidence: high)

| Grade of  recommendation | Benefit vs  risk and burdens | Strength of  supporting evidence |  |
| --- | --- | --- | --- |
| Strong (100%) | Benefits clearly outweigh risks & burdens | High  (metaanalysis data; dose-response gradient) | 1 metaanalysis  45 clinical cases / case series |

**Outcome: survival, severe organ complications**

1. We recommend monitoring for all aspects of renal disease including arterial blood pressure in patients with cobalamin related remethylation disorders. (Quality of the evidence: low)

| Grade of  recommendation | Benefit vs  risk and burdens | Strength of  supporting evidence |  |
| --- | --- | --- | --- |
| Strong (67%) | Benefits clearly outweigh risks & burdens | Low | 128 clinical cases / case series |

**Outcome: survival, severe organ complications**

1. The incidence of vascular complications is significantly reduced with appropriate treatment in late onset patients and may be prevented in early onset patients with remethylation disorders. (Quality of the evidence: moderate)

| Grade of  recommendation | Benefit vs  risk and burdens | Strength of  supporting evidence |  |
| --- | --- | --- | --- |
| Strong (67%) | Benefits clearly outweigh risks & burdens | Moderate  (upgraded for high consistency) | 1 review on late-onset cblC;  and clinical case reports / case series cited therein |

**Outcome: visual and neurocognitive function**

1. As knowledge and awareness of visual dysfunction progression allows timely initiation of appropriate vision intervention programs and support, we recommend that every patient newly diagnosed with a remethylation disorder should receive an ophthalmological consultation independent of the age at diagnosis and severity of disease. (Quality of the evidence: low)

| Grade of  recommendation | Benefit vs  risk and burdens | Strength of  supporting evidence |  | Comment |
| --- | --- | --- | --- | --- |
| Strong (53%) | Benefits clearly outweigh risks & burdens | Low | 98 clinical case reports / case series  Textbook knowledge on visual impairment | Was felt to be very unspecific by some panellists but individual patient’s benefits outweigh the risks and burdens. |

**Outcome: survival, severe organ damage**

1. We suggest that prenatal maternal treatment may be considered in a pregnancy with a fetus with proven cblC disease. (Quality of the evidence: low)

| Grade of  recommendation | Benefit vs  risk and burdens | Strength of  supporting evidence |  |
| --- | --- | --- | --- |
| Weak (74%) | Uncertainty in the estimates of  risks & burdens | Low | 2 case reports |

**Outcome: Survival, severe organ damage; neurocognitive impairment**

1. We strongly recommend immediate treatment with parenteral cobalamin in suspected cases. (Quality of the evidence: moderate)

| Grade of  recommendation | Benefit vs  risk and burdens | Strength of  supporting evidence |  |
| --- | --- | --- | --- |
| Strong (100%) | Benefits clearly outweigh risks & burdens | Moderate  (upgraded for high consistency) | 174 clinical cases / case series |

1. We recommend consideration of betaine treatment as soon as hyperhomocysteinaemia is proven and normal / low methionine confirmed. (Quality of the evidence: moderate)

| Grade of  recommendation | Benefit vs  risk and burdens | Strength of  supporting evidence |  | Comment |
| --- | --- | --- | --- | --- |
| Strong (53%) | Benefits clearly outweigh risks & burdens | Moderate  (upgraded for high consistency) | 174 clinical cases / case series | Intense discussion about risk of betaine treatment, which was considered to weaken the recommendation by some panellists. Others highlighted the benefit of betaine and it’s safety in the presence of normal/low Met |

1. We suggest consideration of additional enteral supplementation with folinic acid or L-methionine in individual cases. (Quality of the evidence: low)

| Grade of  recommendation | Benefit vs  risk and burdens | Strength of  supporting evidence |  |
| --- | --- | --- | --- |
| Weak (63%) | Uncertainty in the estimates of risks & burdens | Low | 174 clinical cases / case series |

**Outcome: survival and severe organ damage**

1. We strongly recommend using parenteral OHCbl in treating patients with the cblC defect and other cobalamin-related remethylation disorders. (Quality of the evidence: high)

| Grade of  recommendation | Benefit vs  risk and burdens | Strength of  supporting evidence |  |
| --- | --- | --- | --- |
| Strong (87%) | Benefits clearly outweigh risks & burdens | High  (upgraded for high consistency) | 128 clinical cases / case series; work on binding of MMACHC protein to OH-Cbl |

1. We recommend applying a starting dose of 1000 µg (1mg) OHCbl daily given parenterally in patients with the cblC defect. This regime has also been applied in other cobalamin-related remethylation defects. (Quality of the evidence: low)

| Grade of  recommendation | Benefit vs  risk and burdens | Strength of  supporting evidence |  | Comment |
| --- | --- | --- | --- | --- |
| Strong (87%) | Benefits clearly outweigh risks & burdens | Low | 128 clinical cases / case series | Although the evidence is low this dose was considered safe by most panellists |

1. We suggest that the minimum effective OHCbl dose and frequency of administration should be individually titrated. Escalating doses of OHCbl may result in biochemical improvement; however, significant clinical benefit remains to be proven. Frequency of administration of OHCbl ranges between daily and weekly without evidence of advantage of one over the other. (Quality of the evidence: low)

| Grade of  recommendation | Benefit vs  risk and burdens | Strength of  supporting evidence |  |
| --- | --- | --- | --- |
| Weak (73%) | Uncertainty in the estimates of  risks & burdens | Low | 2 reviews  128 clinical cases / case series |

**Outcome: survival and severe organ damage**

1. We recommend oral betaine treatment in cblC disease and other cobalamin-related remethylation disorders (Quality of the evidence: low)

| Grade of  recommendation | Benefit vs  risk and burdens | Strength of  supporting evidence |  |
| --- | --- | --- | --- |
| Strong (87%) | Benefits clearly outweigh risks & burdens | Low | 128 clinical cases / case series |

1. We suggest that the minimum effective betaine dose should be individually titrated to improve the levels of tHcy and methionine. (Quality of the evidence: low)

| Grade of  recommendation | Benefit vs  risk and burdens | Strength of  supporting evidence |  |
| --- | --- | --- | --- |
| Weak (40%) | Uncertainty in the estimates of  risks & burdens | Low | 128 clinical cases / case series |

1. Results failed to demonstrate or exclude a beneficial or detrimental effect of folic and/or folinic acid as adjunctive therapy in patients with cblC disease and other cobalamin-related remethylation disorders. (Quality of the evidence: low)

| Grade of  recommendation | Benefit vs  risk and burdens | Strength of  supporting evidence |  |
| --- | --- | --- | --- |
| - | Uncertainty in the estimates of  risks & burdens | Low | 128 clinical cases / case series |

1. Results failed to demonstrate or exclude a beneficial or detrimental effect of oral carnitine as adjunctive therapy in patients with cblC disease and other cobalamin-related remethylation disorders. (Quality of the evidence: low)

| Grade of  recommendation | Benefit vs  risk and burdens | Strength of  supporting evidence |  |
| --- | --- | --- | --- |
| - | Uncertainty in the estimates of  risks & burdens | Low | 128 clinical cases / case series |

**Outcome: survival, severe organ damage; neurocognitive impairment**

1. We strongly recommend not to restrict protein in cblC disease and other remethylation disorders. (Quality of the evidence: moderate)

| Grade of  recommendation | Benefit vs  risk and burdens | Strength of  supporting evidence |  |
| --- | --- | --- | --- |
| Strong (93%) | Benefits clearly outweigh risks & burdens | Moderate  (upgraded for high consistency) | 128 clinical cases / case series; review on feeding in MMA |

1. Met is essential in patients with remethylation defects and we recommend maintaining its plasma levels in the normal range; if necessary this may be achieved by oral methionine supplementation. (Quality of the evidence: low)

| Grade of  recommendation | Benefit vs  risk and burdens | Strength of  supporting evidence |  |
| --- | --- | --- | --- |
| Strong | Benefits clearly outweigh risks & burdens | Low | 174 clinical cases / case series  Textbook knowledge of methionine metabolism |

1. We strongly recommend early treatment with betaine as it improves clinical outcome and prevents neurological deterioration in MTHFR deficiency. (Quality of the evidence: high)

See also recommendation 13

| Grade of  recommendation | Benefit vs  risk and burdens | Strength of  supporting evidence |  |
| --- | --- | --- | --- |
| Strong (100%) | Benefits clearly outweigh risks & burdens | High | 1 metaanalysis  45 clinical cases / case series |

1. Results failed to demonstrate or exclude a beneficial or detrimental effect of folic or folinic acid or 5-CH3THF as adjunctive therapy to restore cellular and cerebral folate deficiency in MTHFR deficiency on clinical outcome. (Quality of the evidence: low).

| Grade of  recommendation | Benefit vs  risk and burdens | Strength of  supporting evidence |  |
| --- | --- | --- | --- |
| - | Uncertainty in the estimates of  risks & burdens | Moderate  (upgraded for high consistency) | 1 metaanalysis  45 clinical cases / case series |

1. We strongly recommend against the use of nitrous oxide in patients with remethylation disorders. (Quality of the evidence: high)

| Grade of  recommendation | Benefit vs  risk and burdens | Strength of  supporting evidence |  |
| --- | --- | --- | --- |
| Strong (90%) | Benefits clearly outweigh risks & burdens | Cases: moderate  (upgraded for high consistency); textbook knowledge: high | 2 case reports; textbook knowledge on NO and methionine synthase |
